# CYP2C9 and OATP1B1 genetic polymorphisms affect the metabolism and transport of glimepiride and gliclazide

**DOI:** 10.1038/s41598-018-29351-4

**Published:** 2018-07-20

**Authors:** Fayou Yang, Xiaomin Xiong, Yonghua Liu, Hong Zhang, Shibo Huang, Yuqing Xiong, Xiao Hu, Chunhua Xia

**Affiliations:** 1 Clinical Pharmacology Institute, Nanchang University, Nanchang, 330006 Republic of China; 2 Basic Department, Jiangxi Health Vocational College, Nanchang, 330201 Republic of China

## Abstract

The therapeutic use of glimepiride and gliclazide shows substantial inter-individual variation in pharmacokinetics and pharmacodynamics in human populations, which might be caused by genetic differences among individuals. The aim of this study was to assess the effect of CYP2C9 and OATP1B1 genetic polymorphisms on the metabolism and transport of glimepiride and gliclazide. The uptake of glimepiride and gliclazide was measured in OATP1B1*1a, *5 and *15-HEK293T cells, and their metabolism was measured using CYP2C9*1, *2 and *3 recombinase by LC-MS. Glimepiride in OATP1B1*1a, *5 and *15-HEK293T cells had V_max_ values of 155 ± 18.7, 80 ± 9.6, and 84.5 ± 8.2 pmol/min/mg, while gliclazide had V_max_ values of 15.7 ± 4.6, 7.2 ± 2.5, and 8.7 ± 2.4 pmol/min/mg, respectively. The clearance of glimepiride and gliclazide in OATP1B1*5 and *15 was significantly reduced compared to the wild-type. Glimepiride in the presence of CYP2C9*1, *2 and *3 recombinase had V_max_ values of 21.58 ± 7.78, 15.69 ± 5.59, and 9.17 ± 3.03 nmol/min/mg protein, while gliclazide had V_max_ values of 15.73 ± 3.11, 10.53 ± 4.06, and 6.21 ± 2.94 nmol/min/mg protein, respectively. The clearance of glimepiride and gliclazide in CYP2C9*2 and *3 was significantly reduced compared to the wild-type. These findings collectively indicate that OATP1B1*5 and *15 and CYP2C9*2 and *3 have a significant effect on the transport and metabolism of glimepiride and gliclazide.

## Introduction

Gliclazide and glimepiride are widely used sulfonylurea hypoglycemic agents that play an important role in the treatment of non-insulin-dependent type 2 diabetes mellitus. The therapeutic use of glimepiride and gliclazide is often complicated by substantial inter-individual variability in their pharmacokinetics and pharmacodynamics in human populations, which might be caused by inter-individual factors, such as genetic polymorphisms of transporters and metabolic enzymes^1^. In previous studies, we found that both glimepiride and gliclazide are substrates of OATP1B1 and are also metabolized by the CYP2C9 enzyme. OATP1B1 and CYP2C9 play an important role in drug disposition and metabolism. Many clinical drugs can be transported from blood into liver mediated by OATP1B1 and then were metabolized by CYP2C9^2,3^. OATP1B1 and CYP2C9 have gene polymorphisms that significantly affect the transport and metabolism of many clinically relevant drugs.

The organic anion transport polypeptide OATP1B1 (also OATP-C or LST-1 gene name *SLCO1B1*) is an uptake transporter that is expressed in the basolateral hepatocyte membrane in the sinusoid^4^, and mainly participates in the transport of endogenous substances (bile acids^5^, thyroid hormones, estrogens^6^) and a variety of clinical drugs (lipid-lowering statin drugs^7^, rifampicin^8^, methotrexate^9^, etc.). It has 2 single nucleotide polymorphisms, 388 A > G (63% mutation frequency in Asian populations) and 521 T > C (16% mutation frequency in Asian populations)^10^, forming four haplotypes, OATP1B1*1a (c.388APc.521 T), OATP1B1*1b (c.388GPc.521 T), OABP1B1*5 (c.388APc.521 C) and OATP1B1*15 (c.388GPc.521 C)^11^. Lots of *in vitro* and *in vivo* studies have revealed that point mutations in OATP1B1*5 and OATP1B1*15 have vital impacts on OATP1B1 transporter activity and have a significant effect on drug transport^12–14^. Nozawa T also reported the genetic polymorphisms could significantly alter the transporting activities of OATP1B1^15^.

CYP2C9 (cytochrome P450 2C9) accounts for about 20% of the total cytochrome P450 in liver microsomes and is the most abundant enzyme in the CYP2 subfamily. Approximately 10–20% of commonly used drugs are metabolized by CYP2C9. The human *CYP2C9* gene that encodes CYP2C9 has genetic polymorphisms that significantly influence drug metabolism^16^. Mutations in the CYP2C9 enzyme lead to differences in enzyme activity, and are an important factor in different individual responses in metabolism by the CYP2C9 enzyme^17^. Numerous *in vitro* and *in vivo* reports have revealed that point mutations in CYP2C9*2 and CYP2C9*3 have vital impacts on CYP enzyme activity and a significant effect on drug metabolism^18–23^.

In the present study we established a transporter and metabolic enzyme *in vitro* model to explore the effects of OATP1B1 and CYP2C9 gene polymorphisms on glimepiride and gliclazide transport and metabolism to establish their role in pharmacokinetic and pharmacodynamic inter-individual variability.

## Materials and Methods

### Chemicals and reagents

Glimepiride (purity 99.8%), gliclazide (purity 99.9%), and gliquidone (purity 99.3%) were supplied by the National Institute for the Control of Pharmaceutical and Biological Products (Beijing, China). Fetal bovine serum (FBS) and Hank’s Balanced Salt Solution (HBSS) was from Solarbio Co., Ltd (Beijing, China). Dulbecco’s modified Eagle’s Medium (DMEM) was from Solarbio Co., Ltd. Methanol and ethyl acetate were from Merck Co. Ltd (Darmstadt, Germany). NADPH, 6-phosphate- glucose, and 6-p-glucose dehydrogenase were purchased from Solarbio Co., Ltd. Human CYP2C9*1, *2 and *3 recombinases (1 nmol/ml P450, 10 mg/ml protein) were provided by the Research Institute for Liver Diseases (Shanghai Corporation Limited, Shanghai, China). All other chemicals and solvents were of the highest grade or analytical grade commercially available.

### Cell line

**Figure 1:**
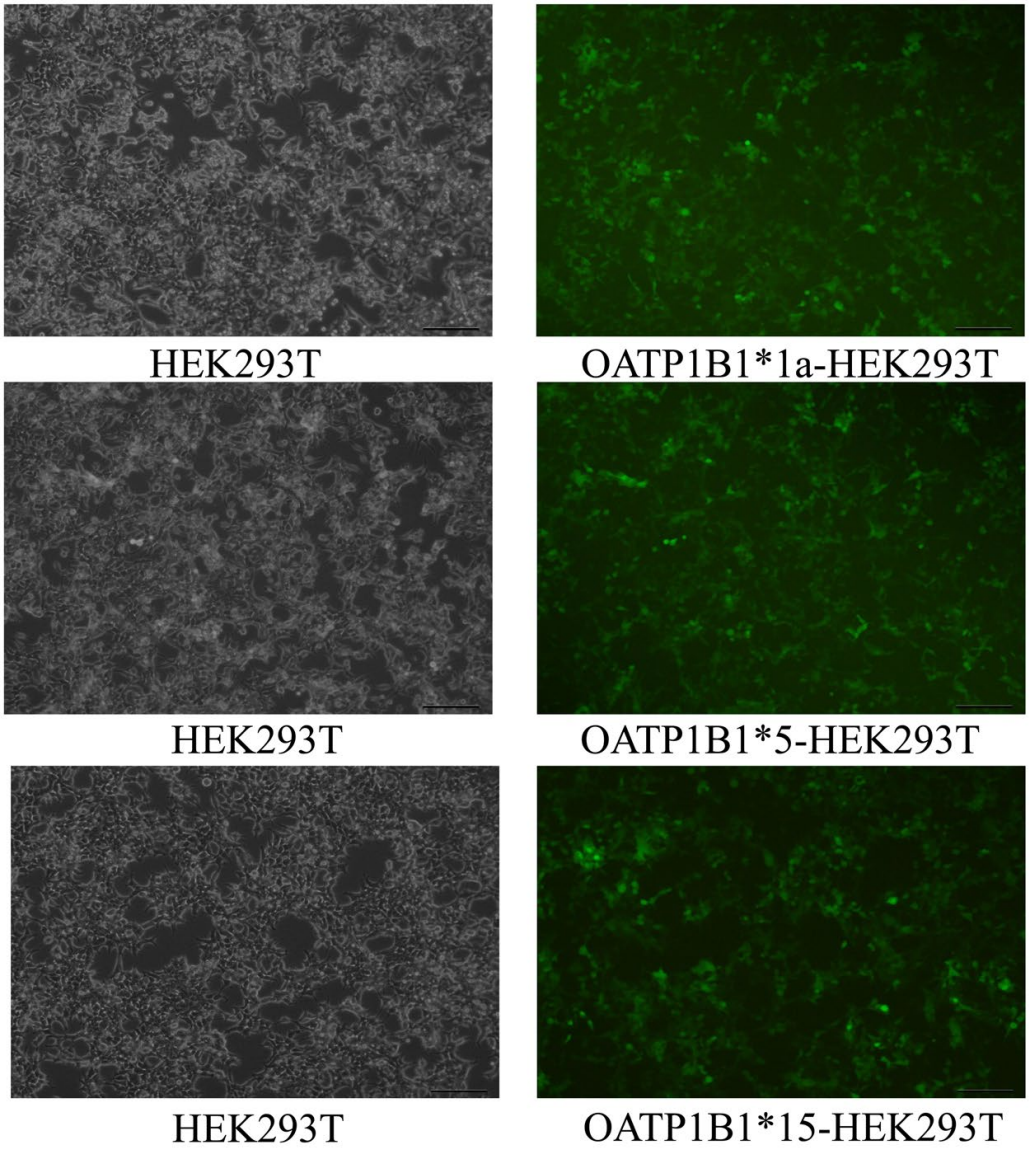
The fluorescent photos of HEK293T cells after being transfected with lentivirus including pGC-FU-OATP1B1*1a, pGC-FU-OATP1B1*5 and pGC-FU-OATP1B1*15.

Human embryonic kidney (HEK293T) cell line was provided by the Chinese Academy of Medical Sciences. OATP1B1*1a, OATP1B1*15, and OATP1B1*5 lentiviral plasmids were purchased from Shanghai Ji Kai Gene Co., Ltd. (Shanghai, China). The HEK293T cell models capable of stably expressing OATP1B1*1a, OATP1B1*15 and OATP1B1*5 were successfully constructed in our study. After being transfected with lentivirus, the green fluorescent protein (GFP) in HEK293T cells was observed using fluorescence microscope (Fig. 1). The HEK293T cells were cultured in high-glucose (4.5 g/L) DMEM with 10% FBS, 100 U/ml penicillin, and 100 μg/ml streptomycin at 37 °C under 5% CO_2_ humidified air.

### Western blot analysis

**Figure 2:**
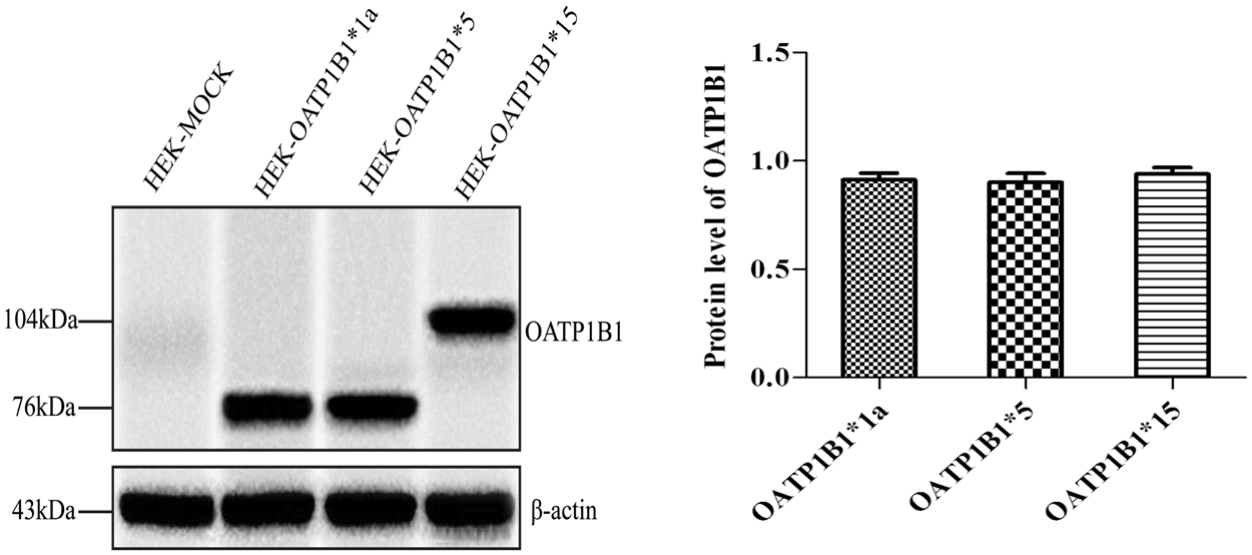
Western blot analysis of OATP1B1 expression in OATP1B1*1a, *5, and *15-HEK293T cells.

The OATP1B1 expression in OATP1B1*1a, *5, *15-HEK293T cells was detected by western blot and the relative protein content of each variant was calculated for standardization of the transport activities (Fig. 2). Cell extracts were prepared in lysis buffer. The cell debris was removed by centrifugation at 12,000 × g at 4 °C for 15 min and the total protein concentration was measured using a BCA Protein Assay Kit (Vazyme Biotech Co., Ltd, Nanjing, China). Protein samples (50 μg) were subjected to SDS-PAGE and electrophoretically transferred to PVDF membranes (EMD Millipore, Bedford, MA). Immunoblots were probed with rabbit polyclonal OATP1B1 antibody (diluted 1:2000) (Sigma Co., Ltd, America) and with mouse polyclonal anti-β-actin (diluted 1:5000) (Sigma Co., Ltd, America) antibody as the loading control. After incubation with HRP-conjugated secondary antibody (Santa Cruz Biotechnology, Inc., Dallas, TX), signals were detected by SuperSignal West Dura (Pierce, Rockford, IL) using a Bio-Rad ChemiDoc XRS imaging system (Bio-Rad Laboratories), and densitometry analysis was performed using Image Lab Software (Bio-Rad Laboratories).

### Quantitative analysis of the uptake of glimepiride and gliclazide in HEK293T cells

The cell density was adjusted to 7.0 × 10^5^/ml, and the cells were added to 12-well culture plates at 1.0 ml per well. For time-course studies, the uptake of glimepiride and gliclazide in HEK293T cells was measured at 2, 5, 10, 15, 20 and 30 min. For concentration dependent studies, the uptake of glimepiride and gliclazide in HEK293T cells were measured in a series of concentrations of 2.5, 5, 10, 20, 40 and 80 μM. The uptake experiments were divided into four groups including MOCK-HEK293T cells group (used as negative control), OATP1B1*1a-HEK293T cells group, OATP1B1*5-HEK293T cells group and OATP1B1*15-HEK293T cells group. Cells were placed in an incubator at 37 °C for 20 min with HBSS containing 10 mM HEPES (HBSS-HEPES, 99:1, v/v). At the end of the incubation period, indicated concentrations of glimepiride or gliclazide were added, cells were placed in the incubator at 37 °C for 15 min or for specified times, and the experiment was terminated by removing the medium and immediately washing cells with ice-cold HBSS four times and then adding 1 ml sterile water. Cells were disrupted by 4 cycles of freeze–thawing at −80 °C, and 200 μl of cell lysate was placed in a 1.5 ml centrifuge tube with 20 μl of 5 μM gliquidone internal standard, 30 μl of glacial acetic acid, and 800 μl ethyl acetate. After vortexing for 5 min, the tube was centrifuged at 15,000 × *g* for 10 min, and 700 μl of the supernatant was concentrated by vacuum drying at 55 °C for 30 min. The resulting concentrate was dissolved in 200 μl of acetonitrile, vortexed for 5 min, and centrifuged at 15,000 × *g* for 10 min; 100 μl of supernatant was used for LC/MS analysis. The protein content of each sample, determined by a BCA Protein Assay Kit (Vazyme Biotech Co., Ltd, Nanjing, China) and the relative quantity of OATP1B1*1a, OATP1B1*5, OATP1B1*15 (determined by western blot) were both used to standardize the effect on transporting activities.

The concentrations of glimepiride and glipizide in cell lysate samples were measured using an LC-MS system equipped with a Shimadzu LC-20AB (Shimadzu Corporation, Kyoto, Japan) coupled to a MS2010EV. Separation was performed using an analytical Shimadzu Pack VP-ODS C18 column (150 × 2.0 mm) with a particle size of 5 μm. The detector voltage was set at +1.85 kV. The curved desolvation line (CDL) and the block heater temperature were set at 250 °C and 200 °C, respectively. The flow rates of drying gas (N_2_) and nebulizing gas (N_2_) were 2.0 L/min and 1.5 L/min, respectively. Mobile phase consisting 0.1% methane acid(A) and acetonitrile (B) at a flowrate of 0.2 ml/min were applied (A:B = 30:70). The analysis was performed in selective ion monitoring mode at [M-H]^−^ *m/z* = 528.25 for gliquidone (internal standard IS) [M-H]^−^ *m/z* = 491.40 for glimepiride, and [M-H]^−^ *m/z* = 324.15 for gliclazide. Glimepiride was found with a retention time of 3.8 min and gliquidone IS with 4.9 min. Gliclazide was found with a retention time of 3.3 min and gliquidone IS with 5.2 min.

### Quantitative analysis of the metabolism of glimepiride and gliclazide by CYP2C9

The reaction mixtures (final volume of 0.2 ml) contained 50 mM sodium phosphate buffer (pH = 7.4) and different genotypes of human recombinant CYP2C9 (CYP2C9*1, CYP2C9*2, and CYP2C9*3 at 0.25 mg/ml). Glimepiride and gliclazide at various concentrations (1–80 μM) were dissolved in methanol, and the final concentration of methanol was no more than 0.5% (v/v). The reactions took place at 37 °C in a shaker. After pre-warming for 5 min, the reaction was initiated by adding a NADPH regenerating system (1.3 mM NADP^+^, 3.3 mM glucose-6-phosphate, 3.3 mM MgCl_2_, and 0.4 U/ml glucose-6-phosphate dehydrogenase). In the negative controls, the NADPH regenerating system was not added. The reactions were stopped at 40 min by adding 100 μl ice-cold acetonitrile. Samples were vortexed and diluted to the proper concentrations, and 20 μl of 5 μM gliquidone (IS) was added to each sample. The samples were then vortexed for 5 min at room temperature. After centrifuged at 15,000 × *g* for 10 min 100 μl of each reconstituted sample was injected for HPLC/MS analysis.

### Statistical analysis

Kinetic parameters were evaluated from suitable curves using GraphPad Prism (GraphPad Software In CA, USA) followed by nonlinear regression analysis. The following equation was used assuming Michaelis–Menten kinetics: V = V_max_ × [S]/([K_m_ + [S]). Where V is the rate of reaction, V_max_ is the maximum velocity, K_m_ is the Michaelis constant (substrate concentration at 0.5 V_max_), and [S] is the substrate concentration. A one-way analysis of variance (ANOVA) was performed to determine whether the differences among all the groups were statistically significant, and then Dunnett’s post hoc test was carried out to statistically compare each experiment group with the wild-type group via SPSS v19.0. The *p* < 0.05 was considered as statistically significant.

## Results

### Uptake characteristics of glimepiride and gliclazide in OATP1B1-HEK293T cells

**Figure 3:**
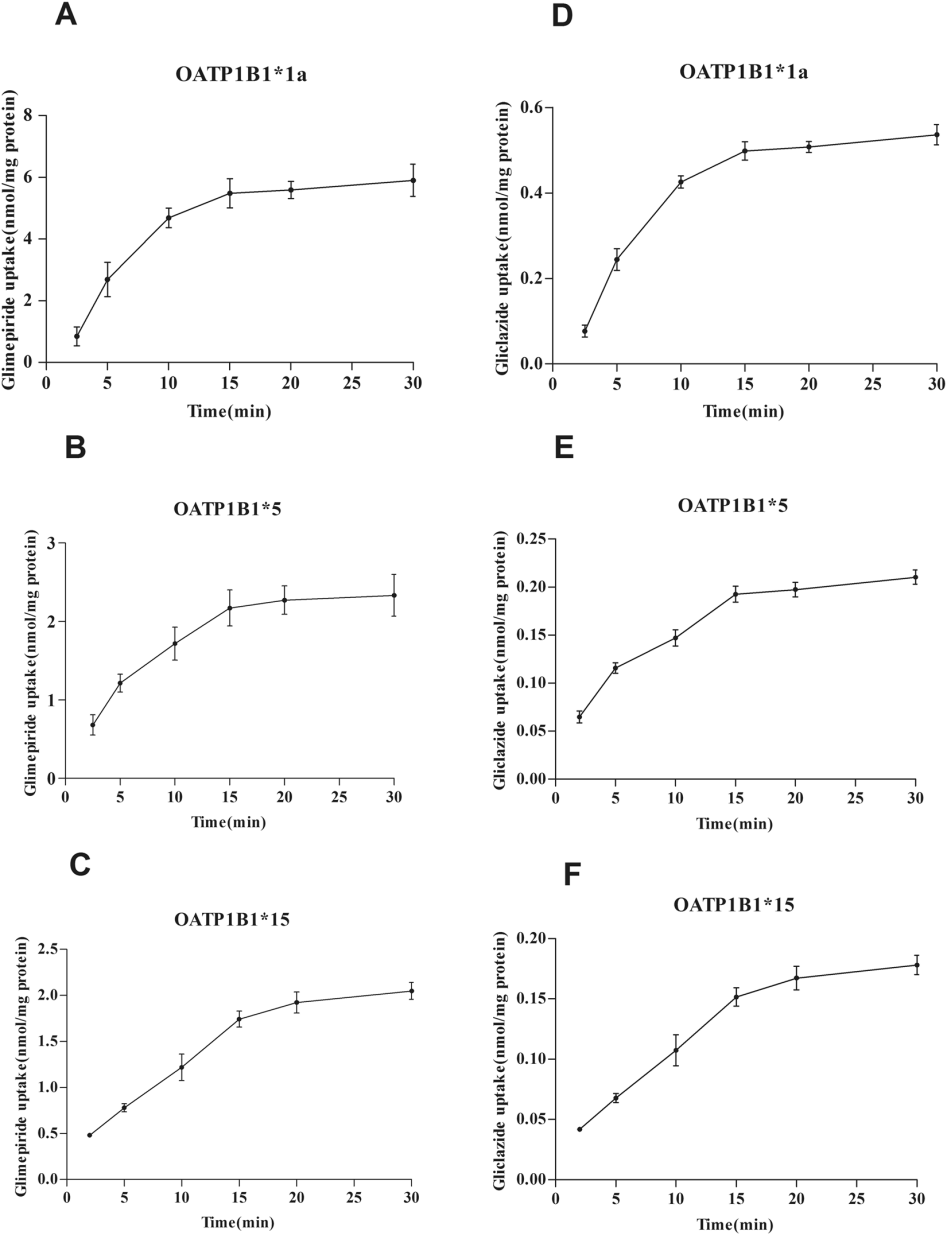
Time-dependent uptake of glimepiride (**A**,**B**,**C**) and gliclazide (**D**,**E**,**F**) in OATP1B1*1a, *5, and *15-HEK293T cells. Forty micromolar glimepiride and gliclazide were incubated with OATP1B1*1a, *5, and *15-HEK293T cells for 2, 5, 10, 15, 20, 30 min. Data points represents the mean ± SD of three separate experiments.

**Figure 4:**
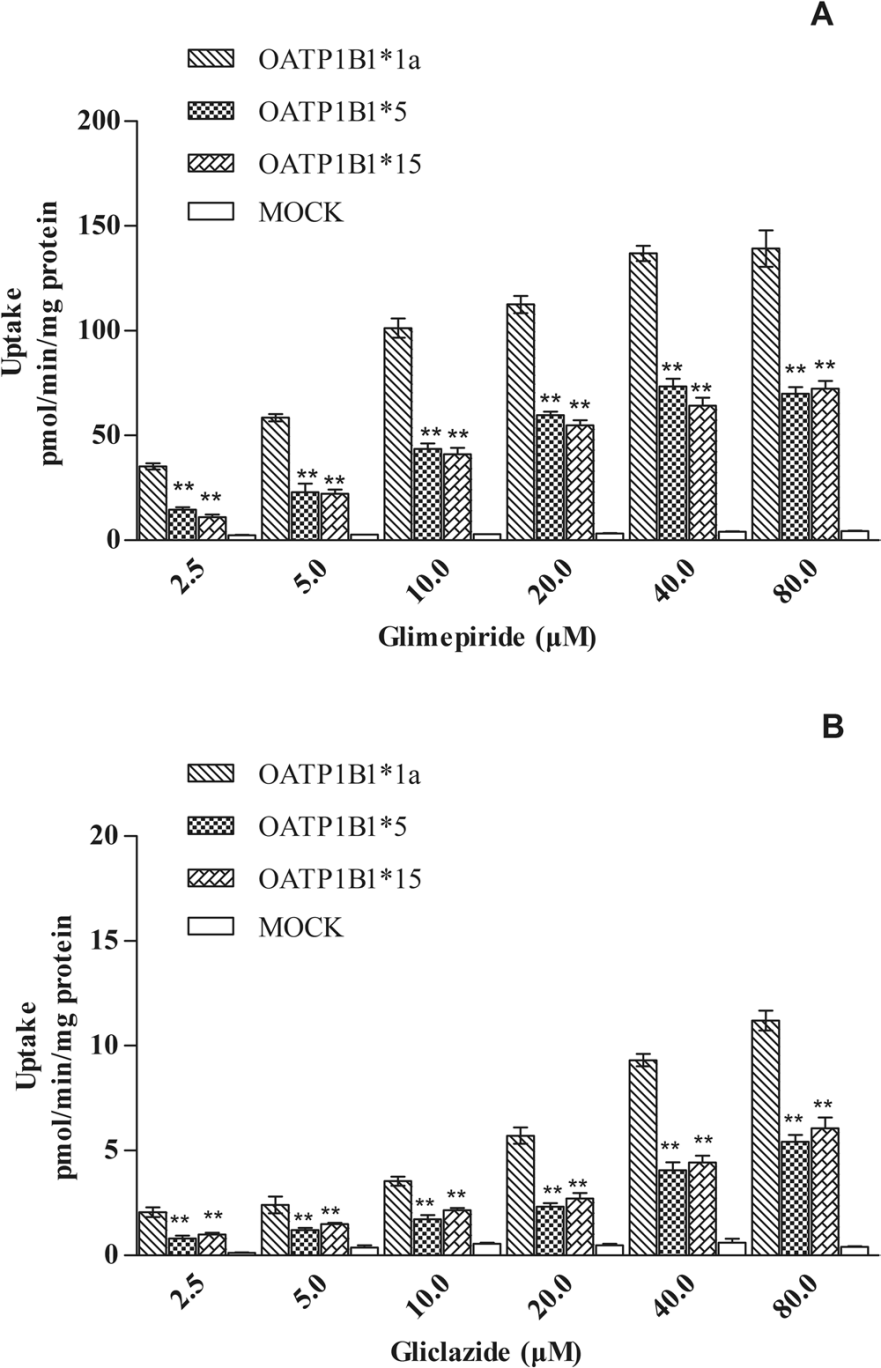
The uptake of glimepiride (**A**) and gliclazide (**B**) in OATP1B1*1a, *5, and *15-HEK293T cells using different substrate concentrations. Uptake of glimepiride and gliclazide in HEK293T cells was studied using concentrations from 2.5 to 80 μM. The uptake experiments were divided into four groups including MOCK-HEK293T cells group, OATP1B1*1a-HEK293T cells group, OATP1B1*5 -HEK293T cells group and OATP1B1*15-HEK293T cells group. Statistical analysis was conducted using SSPS19.0. **Statistically different, P < 0.01, n = 4).

**Figure 5:**
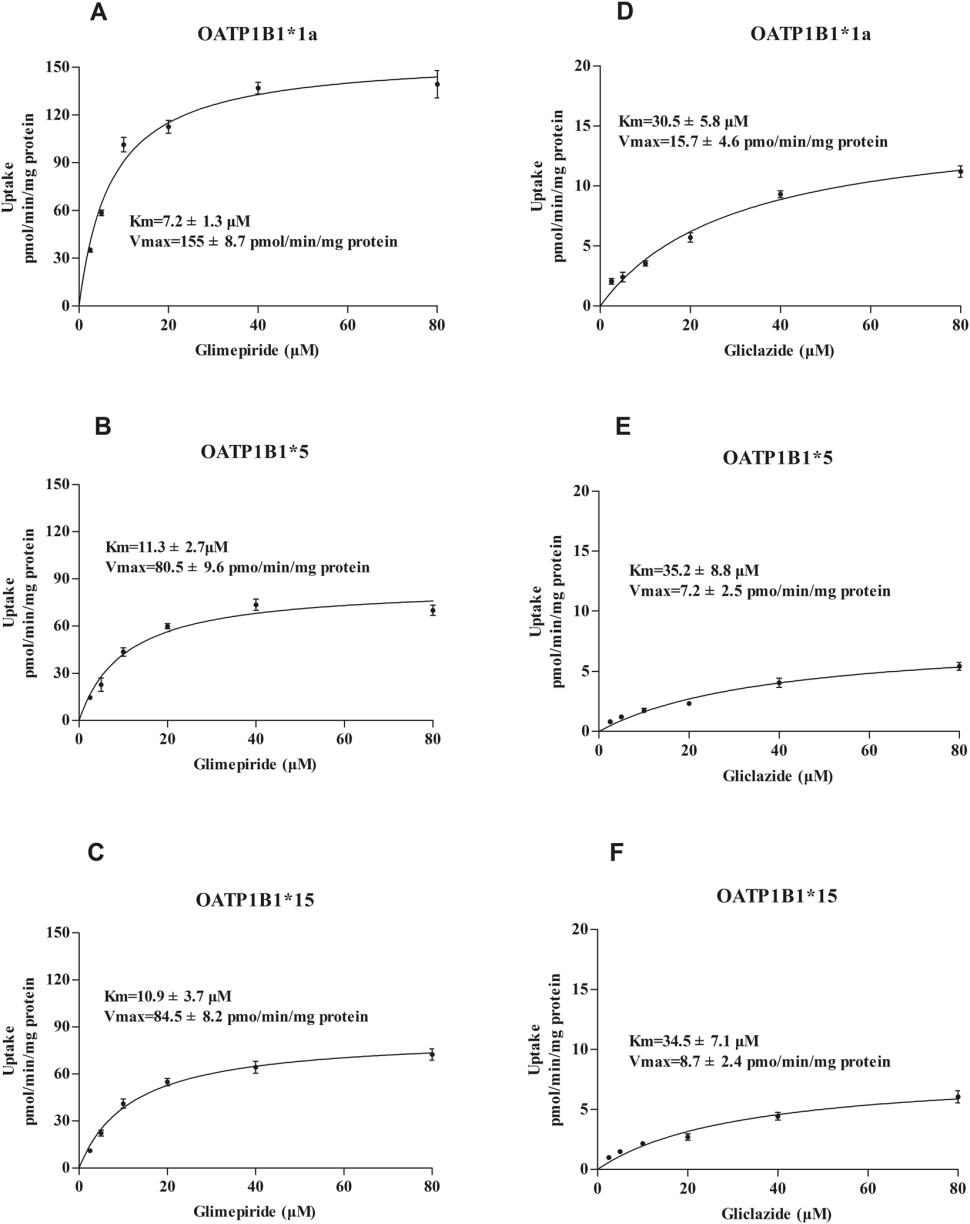
Kinetics of glimepiride (**A**,**B**,**C**) and gliclazide (**D**,**E**,**F**) in OATP1B1*1a, *5, and *15-HEK293T cells. Transport kinetics of OATP1B1 following transient heterologous expression in HEK293T cells conducted for 15 min at varying concentrations of glimepiride and gliclazide (2.5–80 μM). Parameters for saturation kinetics (V_max_ and K_m_) were estimated by nonlinear curve fitting using Prism (GraphPad Software). Data are expressed as mean ± SD (n = 4).

Uptake experiments of glimepiride and gliclazide were carried out as described with the addition of different concentrations of glimepiride and gliclazide (2.5–80 μM) then measured by LC-MS. The uptake time course attained steady-state at 15 min. (Fig. 3). Concentration-dependent experiments showed that the uptake of glimepiride and gliclazide in OATP1B1-HEK293T cells was nearly saturated at 40 μM and was concentration-dependent below that (Figs 4 and 5). The transport of glimepiride and gliclazide by OATP1B1 was related to genotype. Compared with the OATP1B1*1a, mutants OATP1B1*5 and OATP1B1*15 showed significantly decreased transport capacity. The uptake of glimepiride and gliclazide in OATP1B1*5-HEK293T cells and OATP1B1*15-HEK293T cells was significantly less than that of OATP1B1*1a-HEK293T (P < 0.01)) (Fig. 4). For glimepiride, the V_max_ was decreased to 55% and 51%, respectively, in OATP1B1*5-HEK293T and OATP1B1* 15-HEK293T cells compared to OATP1B1*1a-HEK293T cells (Fig. 5A,B,C). The intrinsic clearance (CL_int_, defined as V_max_/K_m_) was decreased to 27% and 30%, respectively, in OATP1B1*5HEK293T and OATP1B1*15-HEK293T cells compared to OATP1B1*1a-HEK293T cells (Fig. 5A,B,C). K_m_ values among the three genotypic groups were not different. For gliclazide, the V_max_ was decreased to 46% and 53%, respectively, in OATP1B1*5-HEK293T and OATP1B1*15HEK293T cells compared to OATP1B1*1a-HEK293T cells (Fig. 5D,E,F). The CL_int_ was decreased to 44% and 50% in OATP1B1*5-HEK293T and OATP1B1*15HEK293T cells compared to OATP1B1*1a-HEK293T cells (Fig. 5D,E,F). K_m_ values among the three genotypic groups were not different.

### Metabolism of glimepiride and gliclazide by recombinant CYP2C9

**Figure 6:**
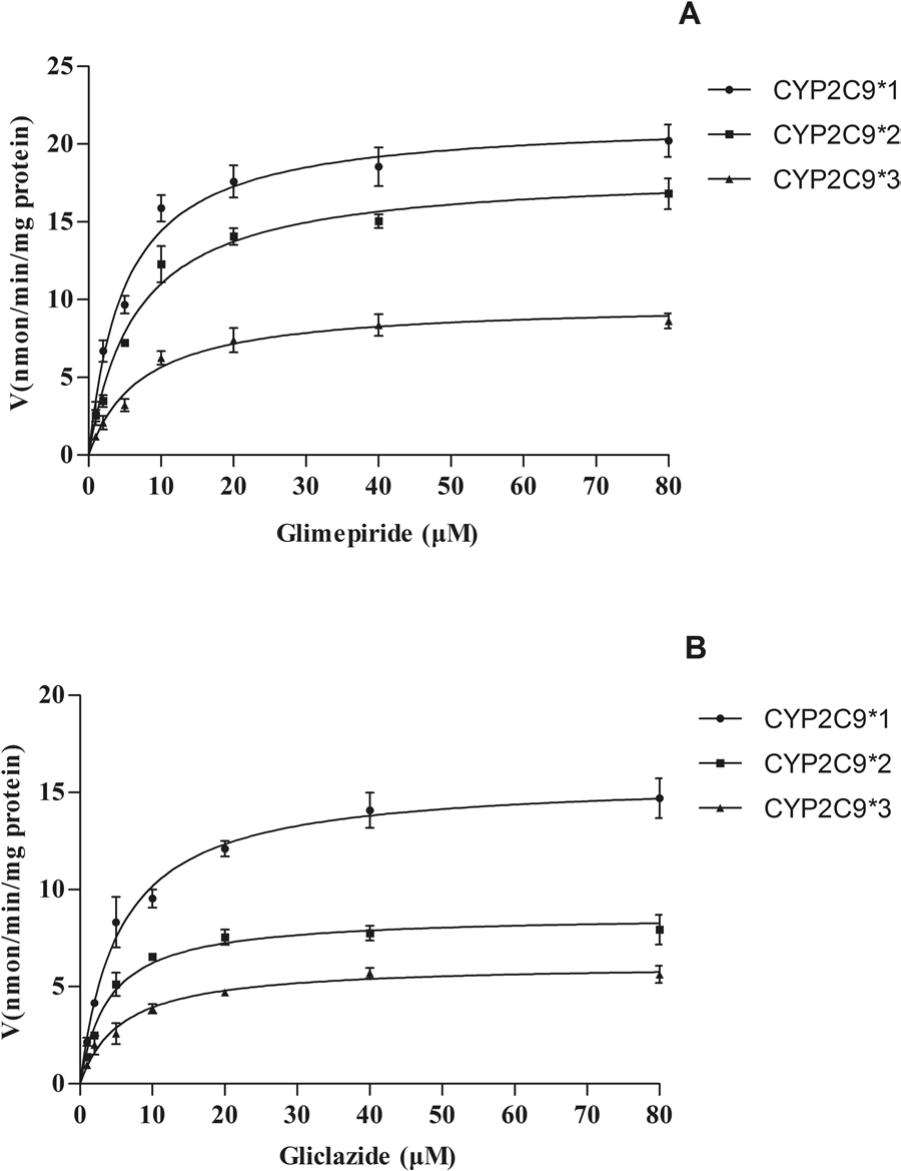
Michaelis–Menten curves of enzymatic activity of recombinant CYP2C9*1,*2, and *3 towards glimepiride (**A**) and gliclazide (**B**). Metabolism of glimepiride and gliclazide in CYP2C9*1,*2, and *3 recombinases were studied using concentrations of 1 to 80 μM. Michaelis–Menten curves of the enzymatic activity of the recombinant wild-type CYP2C9 protein and two variants CYP2C9*2 and *3 towards glimepiride and gliclazide. Data points represent the mean ± SD of three separate experiments.

We assessed the catalytic activities of wild-type CYP2C9*1 and two allelic variants CYP2C9*2 and *3, using glimepiride and gliclazide as substrates. Michaelis–Menten plots for each of the CYP2C9 variants are shown in Fig. 6, and the corresponding kinetic parameters are summarized in Tables 1 and 2. As shown in Tables 1 and 2, the two variants exhibited changed K_m_ and V_max_ values as compared to the wild-type protein. Therefore, the CL_int_ (V_max_/K_m_) values for glimepiride and gliclazide were altered in both of the tested allelic variants. The two variants, CYP2C9*2 and CYP2C9*3, caused the CL_int_ of glimepiride to decrease to 55% and 32%, respectively, compared with the wild-type, and the CL_int_ of gliclazide decreased to 71% and 24%, respectively, compared with the wild-type. These results revealed that point mutations in CYP2C9*2 and CYP2C9*3 have a vital impact on CYP enzyme activity, which has a significant effect on glimepiride and gliclazide metabolism.

**Table 1:** Glimepiride metabolic kinetic parameters in human recombinase CYP2C9*1/*2/*3 (n = 3, mean ± SD).

Protein	K_m_ (μmol/L)	V_max_ (nmol/min/mg)	CL_int_
CYP2C9*1	2.97 ± 1.18	21.58 ± 7.78	7.45 ± 3.02
CYP2C9*2	3.26 ± 1.76	15.69 ± 5.95*	4.11 ± 1.57*
CYP2C9*3	3.70 ± 2.34*	9.17 ± 3.03**	2.42 ± 1.48**

**p < 0.01, *p < 0.05 (*vs*. CYP2C9*1a group).

**Table 2:** Gliclazide metabolic Michaelis–Menten kinetic parameters in human recombinase CYP2C9*1/*2/*3 (n = 3, mean ± SD).

Protein	K_m_ (μmol/L)	V_max_ (nmol/min/mg)	CL_int_
CYP2C9*1	4.87 ± 1.56	15.73 ± 3.11	3.69 ± 2.51
CYP2C9*2	5.18 ± 1.21	10.53 ± 4.06*	2.62 ± 1.89*
CYP2C9*3	6.57 ± 1.82*	6.21 ± 2.94**	0.9 ± 0.21**

**p < 0.01, *p < 0.05 (*vs*. CYP2C9*1a group).

## Discussion

OATP1B1 is an organic anion-transporting polypeptide that plays an important role in transporting certain endogenous and exogenous substrates into the liver. Changes in the transport function of OATP1B1 caused by mutation affect the transfer of substrate drugs, which eventually changes the blood concentration of a drug and affects pharmacological effect. In recent years, OATP1B1 gene polymorphisms in drug transport kinetics, pharmacokinetics and pharmacodynamics have been widely studied^24–26^. *In vivo* observations of the area under the plasma concentration-time curve (AUC) of some clinical drugs (oral antidiabetic drugs and lipid-lowering statin drugs) have been conducted for carriers of haplotypes SLCO1B1*5 or *15 compared to haplotypes SLCO1B1*1a or *1b^12,27–29^, SLCO1B1*5 contains 521 T > C, SLCO1B1*1b contains 388 A > G and SLCO1B1*15 contains both 388 A > G and 521 T > C mutations. Studies have shown that SLCO1B1*1b does not decrease the transport activity of OATP1B1, suggesting that 521 T > C may be the key SNP for determining the functional alteration of OATP1B1^27–29^. In the present study, we found that the uptake of glimepiride in OATP1B1*5 (521 T > C) and OATP1B1*15 (521 T > C, 388 A > G) was significantly reduced compared with OATP1B1*1a, and the uptake kinetic parameters showed the V_max_ and CL_int_ values for glimepiride and gliclazide in OATP1B1*5 and *15 were significantly lower than those for OATP1B1*1a, indicating that the 521 T > C mutation either alone or in a double mutant genotype remarkably impacted OATP1B1 transport activity. Thus, we infer that the uptake of glimepiride and gliclazide are affected primarily by mutation 521 T > C. However, further *in vivo* studies are clearly needed to confirm this since our *in vitro* study may not reflect the activity of OATP1B1 *in vivo*.

It is well-known that metabolic activity changes due to CYP2C9 polymorphisms play an important role in adverse drug reactions. Patients with altered enzymatic activity have a high risk of adverse drug reactions, particularly for CYP2C9-specific substrates such as S-warfarin, phenytoin, glipizide, and tolbutamide, which have narrow therapeutic windows^30^. A study has shown that the oral clearance rate of the sulfonylurea glibenclamide in CYP2C9*3/*3 mutant homozygote subjects is lower by 50% than in CYP2C9*1/*1 wildtype homozygote subjects, and the AUC of CYP2C9*3 heterozygotes is increased by 280% compared with wild-type homozygotes^31^. As for the glimepiride metabolism, a previous *in vitro* study showed that CYP2C9*3 exhibits a lower CL_int_ value (80%) for glimepiride than for the wild-type protein CYP2C9*1^32^. An *in vivo* study has also shown a modest reduction in enzyme activity in healthy volunteers carrying the CYP2C9*2 allele and a strong reduction in those with the CYP2C9*3 allele^33^. However, *in vivo* studies are affected by many other factors, such as physiology, age, and food. Of note, the significant changes in metabolism associated with the CYP2C9*2 and *3 alleles demonstrated *in vitro* and *in vivo* for a variety of substrates do not appear to be consistent for all substrates evaluated. We therefore studied the effect of CYP2C9 gene polymorphisms on drug metabolism through an *in vitro* recombinase enzyme model. We found that CYP2C9*2 was less efficient than CYP2C9*1 in metabolizing glimepiride and gliclazide. However, this was not due to differences in the apparent K_m_, but was the result of a reduced V_max_ and CL_int_ in the metabolism of glimepiride and gliclazide by CYP2C9*2 when compared with CYP2C9*1. Similarly, the allelic variant CYP2C9*3 was also less efficient than CYP2C9*1 in metabolizing glimepiride and gliclazide, The K_m_, V_max_, and CL_int_ were all significantly decreased compared with CYP2C9*1 (Table 1). It is possible that CYP2C9*2 and CYP2C9*3 are risk factors for adverse drug reactions such as hypoglycemia or allergic reactions due to sulphonylurea.

In summary, glimepiride and gliclazide are substrates of OATP1B1 and CYP2C9, and the mutants OATP1B1*5 and *15 can significantly reduce their transport capacity, while CYP 2C9*2 and *3 mutants show significantly reduced metabolism toward glimepiride and gliclazide. It is necessary to simultaneously analyze the transport and metabolic effects of both OATP1B1 and CYP2C9 mutations in clinical settings when using oral hypoglycemic drugs.
